# Development of a Model of Chronic Kidney Disease in the C57BL/6 Mouse with Properties of Progressive Human CKD

**DOI:** 10.1155/2015/172302

**Published:** 2015-05-03

**Authors:** Zahraa Mohammed-Ali, Gaile L. Cruz, Chao Lu, Rachel E. Carlisle, Kaitlyn E. Werner, Kjetil Ask, Jeffrey G. Dickhout

**Affiliations:** ^1^Department of Medicine, Division of Nephrology, McMaster University and St. Joseph's Healthcare Hamilton, Hamilton, ON, Canada L8N 4A6; ^2^Department of Medicine, Division of Respirology, McMaster University and St. Joseph's Healthcare Hamilton, Hamilton, ON, Canada L8N 4A6

## Abstract

Chronic kidney disease (CKD) is a major healthcare problem with increasing prevalence in the population. CKD leads to end stage renal disease and increases the risk of cardiovascular disease. As such, it is important to study the mechanisms underlying CKD progression. To this end, an animal model was developed to allow the testing of new treatment strategies or molecular targets for CKD prevention. Many underlying risk factors result in CKD but the disease itself has common features, including renal interstitial fibrosis, tubular epithelial cell loss through apoptosis, glomerular damage, and renal inflammation. Further, CKD shows differences in prevalence between the genders with premenopausal women being relatively resistant to CKD. We sought to develop and characterize an animal model with these common features of human CKD in the C57BL/6 mouse. Mice of this genetic background have been used to produce transgenic strains that are commercially available. Thus, a CKD model in this strain would allow the testing of the effects of numerous genes on the severity or progression of CKD with minimal cost. This paper describes such a mouse model of CKD utilizing angiotensin II and deoxycorticosterone acetate as inducers.

## 1. Introduction

Although various models of chronic kidney disease (CKD) have been established in the rat [1], the ability to transgenically manipulate the rat is not nearly as well established as in the mouse. Further, many genetic knockout mouse strains, including tissue specific and conditionally inducible knockouts, are available on the C57BL/6 background [2]. However, this mouse has proven to be resistant to the development of CKD. C57BL/6 mice have shown resistance to the induction of CKD by standard techniques such as streptozotocin-induced diabetes [3], bovine serum albumin overload proteinuria [4], and reduced renal mass [5]. Thus, developing a mouse model of CKD on the C57BL/6 background, that shares salient pathological features of human CKD, allows the use of preexisting knockout strains. Experiments on these knockout strains would determine the effect of these genes on the development of renal interstitial fibrosis, proteinuria and the chronic inflammatory response in CKD.

A model has been developed in the C57BL/6 that shows features of progressive human CKD, including proteinuria and inflammation [6]. In this model, mice are uninephrectomized, given Angiotensin (Ang) II infusion and deoxycorticosterone acetate (DOCA) with 1% salt in the drinking water. This model can be referred to as the Ang II/DOCA salt mouse. The use of Ang II and DOCA with a high salt diet in this model results in sodium retention and volume expansion and therefore hypertension [7]. As well, the reduction in renal mass promotes hyperfiltration, which contributes to proteinuria [8]. The dysregulation of the Renin-Angiotensin-Aldosterone system that is stimulated in this model plays a central role in cardiorenal syndrome [9]. This fact is well supported by the success of angiotensin converting enzyme (ACE) inhibitors and angiotensin receptor blockers (ARBs) as first line therapies in the treatment of hypertension and kidney disease patients [9, 10]. As previously demonstrated by Kirchhoff et al. (2008), this mouse model of CKD induces proteinuria and renal injury and increased systolic blood pressure [6]. In this paper, we will further characterize this model by quantifying the apoptotic responses in renal tubular epithelium, as well as renal interstitial fibrosis and the inflammatory response. Further, we sought to determine whether gender alters the severity of the development of these features of CKD in this model.

## 2. Methods and Materials

### 2.1. Model of CKD in C57BL/6 Mice

**Figure 1:**
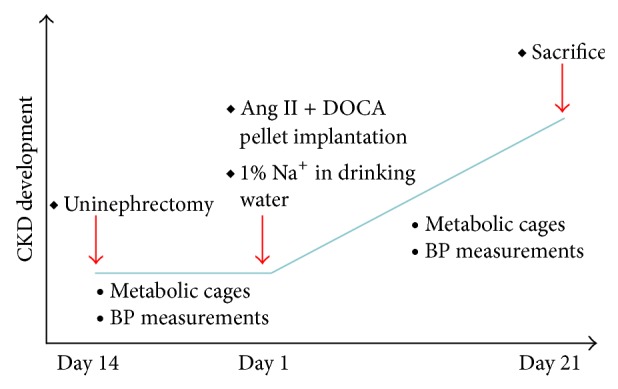
Ang II/DOCA salt model of chronic kidney disease in the C57BL/6 mouse. Graph describing the time course of CKD development.

Ten-week-old mice (gender-balanced groups) underwent uninephrectomy (Unx) or a sham uninephrectomy under isoflurane/oxygen * *anaesthesia 2 weeks before the start of the experiment and were allowed to recover (Figure 1). * *The Unx mice were then given 1% * *sodium chloride in the drinking water and received DOCA pellet implants and Ang II infusion using osmotic minipumps. Model 1004 ALZET osmotic infusion pumps (Durect) containing Ang II in sterile water were subcutaneously implanted in the back of the necks of mice under isoflurane/oxygen anaesthesia to deliver a dose of 1.5 ng Ang II (Sigma) per minute per gram body weight. At this time, a 50 mg 21-day release DOCA pellet (Innovative Research of America, M-121) was also implanted subcutaneously. Mice that underwent the sham Unx were also treated with a sham procedure for subcutaneous implantation. All mice were sacrificed on day 21 after implantation. This animal utilization and the described procedures were approved by the McMaster University Research Ethics Board.

### 2.2. Blood Pressure Measurements

Blood pressure measurements were obtained with tail cuff plethysmography using a CODA (Kent Scientific) blood pressure analyzer before Ang II/DOCA implantation and also before sacrifice (Figure 1). Briefly, animals were placed in restraint and positioned on a heating pad with a tail cuff attached to the machine. The cuff then measured systolic blood pressure, diastolic blood pressure and heart rate.

### 2.3. Urinalysis, Metabolic Cages, and Microalbumin ELISA

Before the surgical procedure and after 3 weeks on treatment with AngII/DOCA salt, mice were placed in metabolic cages for 24 h urine collection (Figure 1). Urine samples were sent to our in-house laboratory to evaluate total protein concentrations and an ELISA was used to measure mouse urine albumin concentration (BETHYL Laboratories) to determine hypertension-induced proteinuria.

### 2.4. Tissue Preparation for Histological Assessment and Immunohistochemical Analysis of Protein Cast Formation, Renal Interstitial Fibrosis, Apoptosis, and Glomerular Sclerosis

Renal tissue was prepared for histological analysis. The tissue was fixed in 4% paraformaldehyde upon sacrificing the animal. The tissue was then embedded in paraffin blocks and sectioned (4 *μ*m) using a microtome. To assess protein cast formation and glomerular injury score, these tissues were stained with Periodic Acid Schiff (PAS) stain and imaged using a light microscope (Olympus).

Collagen deposition indicating extracellular matrix accumulation and renal interstitial fibrosis was evaluated using Masson's trichrome stain (Sigma-Aldrich).

To assess apoptosis, kidney sections were stained using the protocol and reagents provided by the TACS 2 TdT-Fluor* In Situ* Apoptosis Detection Kit (Trevigen, Cat # 4812-30-K). This method is based on specific binding of TdT to 3′-OH ends of DNA and the incorporation of biotinylated deoxyuridine at sites of DNA breaks. This signal is then amplified by avidin-peroxidase, allowing apoptotic cells where DNA fragmentation has occurred, to be visualized with light microscopy [11].

The sections were analyzed using an Olympus BX41 microscope. Immunohistochemistry sections were imaged with 20x and 40x objective lens. For the quantification of protein cast formation, apoptosis, and F4/80 staining, ten microscopic fields were randomly sampled in each of the cortex and the medulla. To score glomeruli, ten microscopic fields were randomly sampled from the cortex allowing the scoring of approximately 50 glomeruli per animal. Images were analyzed for protein cast formation using the MetaMorph program to select and quantify PAS-stained areas as a percentage of the total area of each image. The average of protein cast area density was then calculated for each animal. TUNEL-stained sections were processed using the cell count tool in Image J software. Glomerular sclerosis was assessed based on the scale and method used in a previous study [12].

Lungs from AngII/DOCA salt and sham mice were extracted without performing a bronchoalveolar lavage procedure and fixed in 4% paraformaldehyde for 24 hours. After standard paraffin embedding, 4 *μ*M thick specimens were sectioned and stained with H&E stain to visualize lung damage. Mouse hearts were weighed upon sacrifice and this parameter was normalized to body weight in grams to provide a measure of increase in cardiac mass. The hearts were then fixed in 4% paraformaldehyde for 24 hours, embedded in paraffin, sectioned, and stained with PAS to evaluate areas of hypertrophy.

### 2.5. Statistical Analysis

Statistical analysis was performed using GraphPad Prism software. *t*-tests were used to compare data between groups and significance is denoted by *P* < 0.05. Bar graphs show group averages and standard error of the mean as error bars.

## 3. Results

### 3.1. Development of Hypertensive Proteinuria in Ang II/DOCA Salt Model

**Figure 2:**
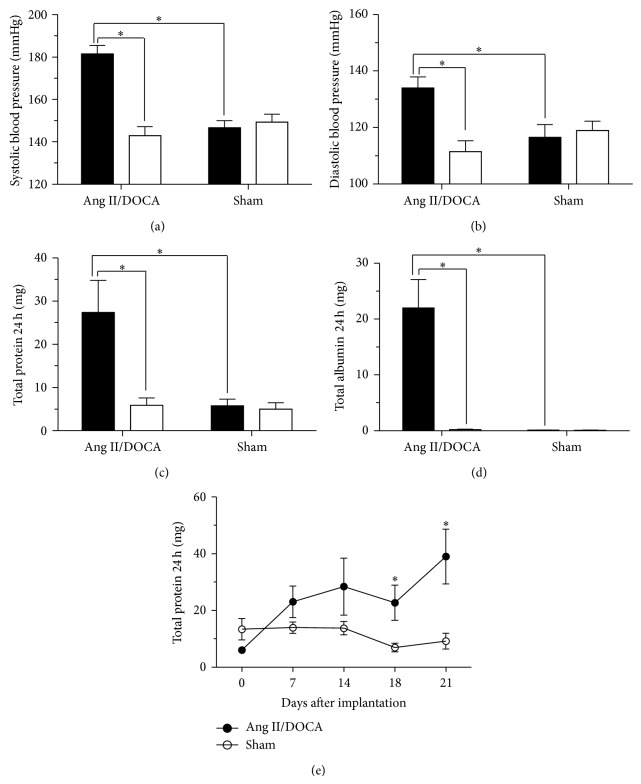
Development of hypertensive proteinuria in Ang II/DOCA salt model of CKD in the C57BL/6 mouse. For all graphs, ∗ indicates a significant difference between two groups where *P* < 0.05. For (a)–(d), □ signifies pretreatment, whereas ■ signifies 21-day posttreatment with Ang II/DOCA. (a), (b) Changes in systolic and diastolic blood pressure in response to Ang II/DOCA. (c), (d) Total 24 h urinary protein and albumin excretion with Ang II/DOCA treatment. (e) Time-course development of proteinuria, expressed as total 24-hour protein excretion at days 7, 14, 18, and 21 posttreatment with Ang II/DOCA.

Blood pressure measurements were taken using tail cuff measurements before the subcutaneous implantation of the osmotic pump containing Ang II and the DOCA pellet as well as at the end of the model before sacrificing the mice. Ang II/DOCA salt mice experienced a significant increase in systolic (Figure 2(a); *P* < 0.001, *N* = 14) and diastolic blood pressure (Figure 2(b); *P* < 0.001, *N* = 14) 21 days after implantation. Ang II/DOCA salt mice also had significantly higher systolic (*P* < 0.001, *N* = 14) and diastolic (*P* < 0.001, *N* = 14) blood pressure compared to sham operated controls 21 days after implantation. This hypertensive response was accompanied by an increase in total protein (Figure 2(c)) and albumin (Figure 2(d)) excreted in the urine over 24 h. Ang II/DOCA salt mice experienced significantly higher total protein (*P* = 0.009, *N* = 14) and total albumin (*P* < 0.001, *N* = 14) in 24 h urine compared to measurements obtained before implantation. Total 24 h urine protein (*P* = 0.006, *N* = 14) and albumin (*P* < 0.001, *N* = 14) were also significantly higher with Ang II/DOCA salt treatment compared to sham controls. In order to assess CKD progression in this model, the time course of the evolution of proteinuria was followed at days 0, 7, 14, 18, and 21 after Ang II/DOCA salt treatment. Proteinuria was significantly elevated at days 18 and 21 (Figure 2(e)).

### 3.2. Characteristics of Renal Tissue Damage in Response to Ang II/DOCA Salt Treatment

**Figure 3:**
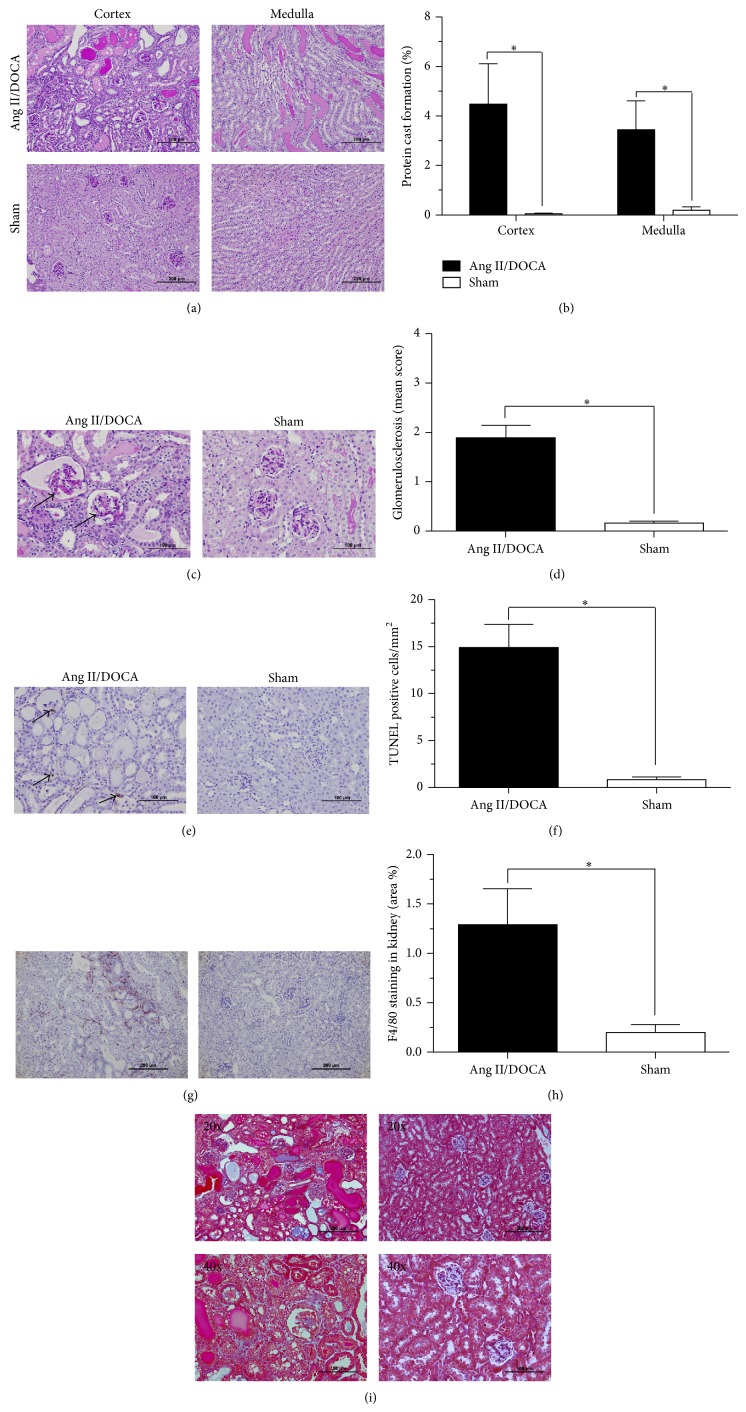
Renal tissue damage in response to Ang II/DOCA salt CKD mouse model. For all graphs, ∗ indicates a significant difference between two groups where *P* < 0.05. Effect of Ang II/DOCA salt model on (a), (b) protein cast formation in the cortex and medulla, (c), (d) glomerulosclerosis, (e), (f) apoptosis, (g), (h) macrophage (F4/80+ cells) infiltration, and (i) interstitial fibrosis (Masson's trichrome staining).

The proteinuria data was consistent with the immunohistological analysis of PAS-stained kidney sections showing the percentage of protein cast formation as compared to sham controls (Figure 3(a)). Kidneys from Ang II/DOCA salt mice showed a significantly higher percentage of protein cast formation as compared to sham controls. PAS staining images show increased protein cast formation in renal tubules of the cortex (*P* = 0.003, *N* = 14) and medulla (*P* = 0.01, *N* = 14) in Ang/II/DOCA salt mice compared to sham controls (Figure 3(b)). Higher magnification images of PAS staining showed increased glomerular sclerosis in the cortex of Ang II/DOCA salt mice compared to sham mice as indicated by the arrows (Figure 3(c)). Quantification of glomerular sclerosis by two independent assessors utilizing the method of Raij et al. [12] indicated that the Ang II/DOCA salt mice displayed significantly elevated glomerular sclerosis scores (*P* < 0.001, *N* = 10) compared to age-matched sham operated controls (Figure 3(d)).

In order to assess renal cell loss, we performed TUNEL staining. TUNEL staining images demonstrated increased apoptosis (Figure 3(e), arrows) in kidney micrographs of Ang II/DOCA salt mice compared to sham animals (Figure 3(f); *P* < 0.001, *N* = 10). Immunohistochemical staining for F4/80, a highly specific macrophage cell surface marker [13], demonstrated a significant increase in macrophage infiltration density (Figure 3(g)) in response to Ang II/DOCA salt treatment compared to sham controls (Figure 3(h); *P* = 0.02, *N* = 5). To examine renal interstitial fibrosis, we stained kidney sections with Masson's trichrome stain. Indeed, in our Ang II/DOCA salt model, we saw increased collagen deposition as indicated by blue-stained fibres in the renal interstitium of areas showing kidney damage in the Ang II/DOCA salt mice compared to sham controls (Figure 3(i)).

### 3.3. Impact of CKD on Cardiac and Lung Function and Morphology

**Figure 4:**
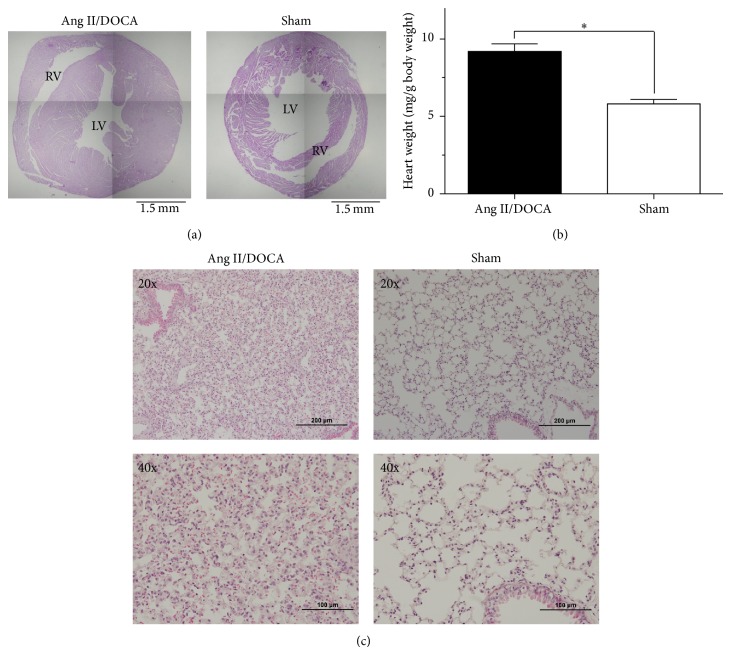
Effect of Ang II/DOCA salt model on cardiac and lung tissue. (a) Images of heart cross-section cardiac hypertrophy in Ang II/DOCA salt model. (b) Graph showing increase in heart weights (mg/g of body weight) in response to Ang II/DOCA where ∗ indicates a significant difference between two groups (*P* < 0.05). (c) Lung damage (20x, 40x) in response to Ang II/DOCA compared to SHAM controls.

Microscope images of heart cross-section portrayed right and left ventricle hypertrophy in response to Ang II/DOCA salt treatment (Figure 4(a)). Cardiac muscle hypertrophy is an adaption to fluid retention and hypervolemia resulting from the model. This phenomenon is confirmed by the increase in heart weights (mg/g of body weight) (*P* < 0.001, *N* = 14) observed in mice treated with Ang II/DOCA salt compared to sham controls (Figure 4(b)). Lung sections derived from Ang II/DOCA salt mice showed features of pulmonary edema, inflammatory infiltrates, and thickening of the alveoli. Signs of increased vessel thickness and capillaries congested with red blood cells are also observed (Figure 4(c)). Although further characterization is required, it is likely that the observed pulmonary changes are driven by sodium retention and the severe hypervolemic changes observed in this model.

### 3.4. Impact of Gender on Renal Tissue Damage Induced by Ang II/DOCA Salt Model

**Figure 5:**
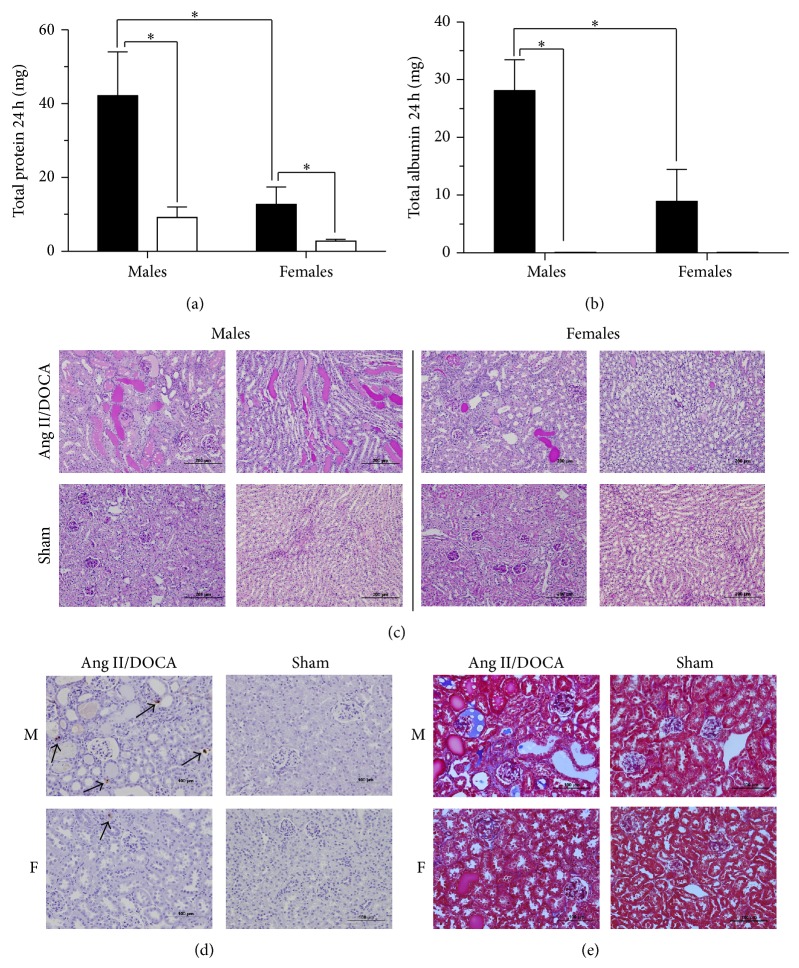
Impact of gender on CKD induced by Ang II/DOCA salt model. For all graphs, ∗ indicates a significant difference between two groups where *P* < 0.05. Ang II/DOCA salt is denoted by ■, whereas □ denotes SHAM operated controls. “M” and “F” indicate sections from male and female mouse kidneys, respectively. Influence of gender on the development of (a) proteinuria, (b) albuminuria, (c) protein cast formation, (d) apoptosis, and (e) interstitial fibrosis.

Both male (*P* = 0.02, *N* = 7) and female (*P* = 0.04, *N* = 7) Ang II/DOCA salt mice developed proteinuria compared to their respective sham controls as a result of the CKD model. However, male Ang II/DOCA salt mice showed significantly higher total protein in 24 h urine (*P* = 0.04, *N* = 7) compared to female Ang II/DOCA salt mice (Figure 5(a)). Total 24 h urine albumin measurements showed a similar trend where male Ang II/DOCA salt mice (*P* < 0.001, *N* = 6) experience a higher level of albumin in the urine compared to their sham controls and compared to female Ang II/DOCA salt mice (*P* = 0.03, *N* = 6). Although female Ang II/DOCA salt mice had a higher albumin level in 24 h urine (*P* = 0.11, *N* = 6) than their sham controls, this increase was not significant (Figure 5(b)). Protein cast formation was significantly higher in the cortex (*P* = 0.02, *N* = 7) and medulla (*P* = 0.04, *N* = 7) of Ang II/DOCA salt male mice and in the cortex (*P* = 0.004, *N* = 7) and medulla (*P* = 0.01, *N* = 7) of Ang II/DOCA salt female mice compared to their respective sham controls. The percentage of PAS-stained area was higher in the cortex (*P* = 0.048, *N* = 7) and medulla (*P* = 0.145, *N* = 7) of male mice treated with Ang II/DOCA salt compared to female mice treated with Ang II/DOCA salt (Figure 5(c)). TUNEL staining was used to assess the influence of gender on kidney cell death and, ultimately, nephron loss. Although female Ang II/DOCA salt mice experienced an increase in apoptosis (*P* = 0.03, *N* = 5) compared to female sham controls, the apoptosis observed in male mice treated with Ang II/DOCA salt was significantly higher (*P* = 0.002, *N* = 5) compared to Ang II/DOCA salt treated females (Figure 5(d)). In addition, Masson's trichrome staining showed a higher level of collagen deposition and renal interstitial fibrosis (indicated by blue staining) in male Ang II/DOCA salt mice compared to female Ang II/DOCA salt mice (Figure 5(e)).

## 4. Discussion

CKD is characterized by reduction in glomerular filtration rate (GFR), albuminuria, and structural or functional abnormalities of the kidney [14]. CKD is increasing in prevalence globally and its comorbidities include cardiovascular disease, increased all-cause and cardiovascular mortality, kidney disease progression to end stage renal disease (ESRD), and acute kidney injury [15, 16]. The financial impact of CKD places a large burden on health care systems with high costs associated with renal replacement therapy, dialysis, and cardiovascular complications [15]. The C57BL/6 mouse has been the most preferred strain for the generation of transgenic and knockout animal models and will be utilized to develop a genome-wide panel of knockout animals to characterize gene function [2]. The Ang II/DOCA salt model described here shows a robust CKD response that closely mimics human CKD. Developing this model allows the use of genetically modified mice in order to identify gene targets that play a role in CKD development and progression.

Similar to the description of the model provided by Kirchhoff et al. [6], we found systolic blood pressure to be elevated; additionally, we determined that diastolic blood pressure was also elevated. We present data for the total 24-hour excretion of both protein and albumin in the urine, demonstrating that the Ang II/DOCA salt model induced significant proteinuria and albuminuria. This finding is similar to the increase in albuminuria/creatinine ratio described by Kirchhoff et al. [6]. Further, we show the evolution of proteinuria in the model at days 0, 7, 14, 18, and 21 where we observe a statistically significant increase in proteinuria at day 18 and day 21. We also demonstrated that protein cast formation and glomerular sclerosis were significantly increased in the model, similar to findings by Kirchhoff et al. [6]. We further investigated renal injury by examining apoptosis through TUNEL staining and found it to be significantly elevated. Additionally, the kidneys were found to have a significant infiltration of macrophages and showed renal interstitial fibrosis through trichrome staining. Similar to Kirchhoff et al. [6], we found end-organ damage in the heart characterized by cardiac hypertrophy. We extended these findings to examine the lung, where we noted an edematous effusion in airspaces. Further, we characterized the model for gender differences and found that the female gender imparted protection from proteinuria, albuminuria, protein cast formation, apoptosis and renal interstitial fibrosis.

Hypertensive proteinuria is a common feature of CKD. Therefore, animal models that display this feature have been used to investigate disease-specific mechanisms, molecular pathogenesis and potential therapies [1]. The increased glomerular permeability during hypertensive CKD allows protein hyperfiltration into the proximal tubules causing renal tissue damage. Filtered albumin and other proteins that accumulate within intracellular compartments of proximal tubular cells perturb cell function by several mechanisms [17]. Hypertension is an important contributor to ESRD [18]. Proteinuria is associated with glomerular damage and podocyte depletion [19] and can be used in the classification of different stages of CKD clinically [20]. The Ang II/DOCA salt model displays hypertensive proteinuria that mirrors human CKD, as demonstrated by a significant increase in systolic and diastolic blood pressure, as well as proteinuria and albuminuria. Overt proteinuria evolved later in this model at days 18 and 21, indicating disease progression in response to glomerular injury. The glomerulosclerosis seen in our model involves inflammation and fibrosis around the area of the damaged glomeruli and could potentially result in tubular atrophy and degeneration [19]. Tubulointerstitial injury caused by proteinuria includes the formation of protein casts that may block the tubular lumen [19] and this phenomenon has been shown in our model.

As CKD progresses tubular cell atrophy results primarily due to apoptosis. An increase in apoptosis has been demonstrated using TUNEL staining on kidney sections from diabetic nephropathy patients [21] and from rats in the streptozotocin-induced diabetic nephropathy model [22]. In addition, apoptotic nuclei have been observed in polycystic human kidneys and kidney sections from mouse models of the disease [23]. The loss of renal tubular epithelial cells through apoptosis occurs in both acute and chronic kidney diseases [24]. Apoptotic cell loss from nephron segments leads to tubular atrophy and their loss eventually leads to a decline in GFR. Since the Ang II/DOCA salt model displays a robust apoptotic response, it could be used in identifying therapeutic targets against apoptosis that are able to halt CKD progression [24].

Macrophage infiltration has been studied as a universal feature of tubulointerstitial damage regardless of disease origin [25, 26] and is a key feature of experimental and human kidney disease models [26, 27]. Macrophages have been shown to release cytotoxic moieties such as proteolytic enzymes, reactive oxygen, and nitrogen species, as well as proinflammatory cytokines and chemokines [28]. By expressing cytokines such as TGF-*β* and connective tissue growth factor, macrophages are able to induce myofibroblast differentiation and extra cellular matrix deposition, key processes in renal interstitial fibrosis [29, 30]. Increased macrophage infiltration is associated with glomerulosclerosis and tubulointerstitial fibrosis [27, 31, 32]. Both macrophage infiltration and renal interstitial fibrosis have been demonstrated in the Ang II/DOCA salt model of CKD. Further studies would be focused on the time course of the development of these phenomena in order to establish pathways that lead to CKD progression.

CKD, through its effects on volume overload, plays an important pathophysiological role in multiple organ failure. The effect of volume overload on the heart is to develop cardiac hypertrophy through myocardial stretch. This effect is reflected in our model. As hypertrophy progresses from compensated to decompensated heart failure, volume overload affects the lungs and results in pulmonary edema [33]. This phenomenon is also reflected in our model.

The findings that Ang II/DOCA salt mice show a gender-based difference in severity of CKD are consistent with findings in the human population [34] and in agreement with previous clinical and experimental studies on the protective effect of female gender on the development of renal diseases [35–38]. Female mice, subjected to this model, show significantly lower levels of proteinuria. Ang II/DOCA salt female mice also experienced a trend for decreased protein cast formation and collagen deposition, thereby indicating a lower level of renal tissue damage compared to Ang II/DOCA salt male mice. A recent study identified gender difference as a modifier of susceptibility to ER stress-induced injury in tunicamycin-treated mice, a model of acute kidney injury, showing reduced renal pathology in female mice [39]. The reduced induction of apoptosis in female Ang II/DOCA salt mice in our CKD model is therefore in agreement with* in vivo* experiments of endoplasmic reticulum stress-induced acute kidney injury in the tunicamycin mouse models [39].

Clinical studies have demonstrated an association between male gender and a faster rate of CKD progression [36]. Interestingly, the impact of gender is restricted to premenopausal women and is, therefore, thought to be an estradiol-mediated protective effect [35]. This finding is supported by experimental studies. Aged male rats have been shown to develop decreased GFR, increased glomerular injury, and increased proteinuria earlier than female rats [37]. In addition, estradiol treatment caused a reduction in glomerulosclerosis, expression of adhesion, and extracellular matrix molecules and prevented tubular damage in animal models of unilateral nephrectomy and chronic renal allograft rejection [38, 40]. These studies indicate a renoprotective role for estradiol and although our experiments show that female gender imparts a protective effect on CKD development, the potential role of estradiol is still to be determined.

In conclusion, the Ang II/DOCA salt model produces a significant degree of CKD that mimics progressive human CKD in the C57BL/6 mouse. This pathology includes proteinuria, intertubular protein casts, renal interstitial fibrosis, and tubular epithelial cell apoptosis. As this model recapitulates many of the aspects of pathology found in human CKD [41], it will be of great utility to determine the specific effect of various candidate genes on CKD severity and progression. Further, a gender effect was observed where female mice showed lower proteinuria and apoptosis in the CKD model. Future work in this model may allow the precise molecular nature of the protective effect of the female gender to be determined.
